# Design for Additive Manufacturing and Investigation of Surface-Based Lattice Structures for Buckling Properties Using Experimental and Finite Element Methods

**DOI:** 10.3390/ma15114037

**Published:** 2022-06-06

**Authors:** Gul Jamil Shah, Aamer Nazir, Shang-Chih Lin, Jeng-Ywan Jeng

**Affiliations:** 1 Department of Mechanical Engineering, National Taiwan University of Science and Technology, No. 43, Section 4, Keelung Road, Taipei 106, Taiwan; guljamilshah@gmail.com; 2 High Speed 3D Printing Research Center, National Taiwan University of Science and Technology, No. 43, Section 4, Keelung Road, Taipei 106, Taiwan; xyz@mail.ntust.edu.tw; 3 Department of Industrial and Systems Engineering, The Hong Kong Polytechnic University, Hung Hom, Kowloon, Hong Kong SAR 999077, China; 4 Graduate Institute of Biomedical Engineering, National Taiwan University of Science and Technology, Taipei 106, Taiwan

**Keywords:** additive manufacturing, buckling behavior, surface-based lattice structures, critical buckling load, TPMS, FEA

## Abstract

Additive Manufacturing (AM) is rapidly evolving due to its unlimited design freedom to fabricate complex and intricate light-weight geometries with the use of lattice structure that have potential applications including construction, aerospace and biomedical applications, where mechanical properties are the prime focus. Buckling instability in lattice structures is one of the main failure mechanisms that can lead to major failure in structural applications that are subjected to compressive loads, but it has yet to be fully explored. This study aims to investigate the effect of surface-based lattice structure topologies and structured column height on the critical buckling load of lattice structured columns. Four different triply periodic minimal surface (TPMS) lattice topologies were selected and three design configurations (unit cells in *x*, *y*, *z* axis), i.e., 2 × 2 × 4, 2 × 2 × 8 and 2 × 2 × 16 column, for each structure were designed followed by printing using HP MultiJet fusion. Uni-axial compression testing was performed to study the variation in critical buckling load due to change in unit cell topology and column height. The results revealed that the structured column possessing Diamond structures shows the highest critical buckling load followed by Neovius and Gyroid structures, whereas the Schwarz-P unit cell showed least resistance to buckling among the unit cells analyzed in this study. In addition to that, the Diamond design showed a uniform decrease in critical buckling load with a column height maximum of 5193 N, which makes it better for applications in which the column’s height is relatively higher while the Schwarz-P design showed advantages for low height column maximum of 2271 N. Overall, the variations of unit cell morphologies greatly affect the critical buckling load and permits the researchers to select different lattice structures for various applications as per load/stiffness requirement with different height and dimensions. Experimental results were validated by finite element analysis (FEA), which showed same patterns of buckling while the numerical values of critical buckling load show the variation to be up to 10%.

## 1. Introduction

Additive manufacturing (AM) has gained interest due to its ability to fabricate complex geometry [1] in lesser time [2] with less material processing [3] and allows the manufacture of TPMS structures, which cannot be fabricated by traditional manufacturing [4]. The investigation of lattice structures fabricated by AM for buckling strength is the active research area as buckling is a critical failure mechanism that results in unstable structures [5,6]. Different studies have concluded that surface-based lattice structures have good combination of specific stiffness and axisymmetric stiffness, high surface volume ratio and pore connectivity [7] and provide their advantages in applications where buckling is the integral failure mechanism. This enabled the design and fabrication of different structures to obtain high performance with variations in design parameters [8,9,10].

In Lattice structures, buckling is one of the important parameters that lead to the failure of the structure when subject to compressive loads. Lattice structures are a better choice for applications where stiffness is required to be as high as possible for a given mass [6]. Despite the promising qualities of lattice structures, stress concentration at the joints resulted in a considerable drop in their mechanical performance [11], which can lead to yield, creep, damage, or premature failure [12,13]. Excessive axial load on columns causes buckling, which is a common failure mode of lattice structures [14].

Surface-based lattice structures showed unique stress and volume distributions due to which it exhibited superior mechanical performance, e.g., higher energy absorption capacity, compressive strength and elasticity modulus compared to strut-based lattices [15]. Alabort et al. used different size and volume fraction of TPMS for fabrication of bone tissues, and the results revealed that stiffness and yield strength of the designed lattices match a wide range of bone types [16]. TPMS potentially offered improved properties over strut-based lattice structures for bone implant applications. Maskery et al. analyzed Gyroid with cell sizes of 3 mm, 4.5 mm, 6 mm, and 9 mm and found that one should choose a small cell size to avoid low-strain structural failure, which occurred due to localized fracture and crack propagation. In addition, he also found that Gyroids have almost three-times greater specific energy absorption (SEA) than body-centered-cubic (BCC) structures with similar porosity [17]. Zhao et al. [15] studied the impact of local geometric features on compressive mechanical failure, using relative densities of 10%, 20% and 30%, and he found that the failure mechanism of the TPMS-based samples with a high volume fraction changed to brittle failure observed by scanning electron microscope (SEM), as their struts were more affected by the axial force and fractured on struts.

Major engineering failure such as premature failure and crack growth may be caused by improper design. Therefore, before the subjection of lattice structure to the application, it is very important to find the optimal parameters. Nazir et al. analyzed buckling behavior of strut-based lattice column and revealed that the critical buckling load depended on the shape and size, diameter, distribution of mass and position of vertical beam and the count of inclined as well as horizontal beams [5]. In another study, Nazir et al. analyzed the effect of unit cell size and column height on critical buckling load strut-based lattice column and found that critical buckling load increases with the increase in unit cell size or decrease in cellular column height, additionally, the failure of cellular columns having larger height-to-width (h/w) ratios happens due to global buckling, whereas local bucking dominates for smaller h/w ratios [6]. Maskery et al. designed five different surface-based lattice structures functionally graded by tailored volume friction and examined elastic moduli. The result revealed that the I-WP lattice structure recorded the highest stiffness in one loading direction. However, the Diamond structure also showed greater isotropic behavior [18]. The unidirectional isotropic strut that is built of porous matter is analyzed by Magnucki et al. [19] and the systematic solution for the calculation of the critical buckling load of the beam was obtained. The bending and buckling behavior of a porous plate subjected to uniformly distributed force and buckling force was studied by Magnucka-Blandzi [20] and the critical load linearly decreased with the increased porosity of the plate. Overvelde et al. investigated two-dimensional soft porous lattice for the influence of structure shapes on buckling and reported that the structure’s design affects the buckling actions of the soft, porous system [21]. Saghaian et al. [22] systematically investigated three various TPMS designs with constant porosity levels and found that the mechanical properties of porous samples were highly dependent on the structure’s geometry. Additionally, the author claims that Gyroid and Diamond structures are suitable for high strain, while the Schwartz-P structure was recommended for low-stress levels. The buckling analysis of functionally graded porous plate was studied in another study to explore the effects of the lattice’s structure shape, different boundary conditions, and plate thickness, and it was found that the porosity coefficients play significant influences on the buckling behavior and reliability of the structures [23]. Kadkhodapour et al. [24] studied the failure mechanism of I-WP and F-RD TPMS structures using 10 mm × 10 mm × 10 mm cubic cell size composed of 5 × 5 × 5 unit cells. They observed bilateral layer-by-layer failure due to the buckling of micro-struts in I-WP while F-RD-type failed due to global shearing bands. They reported the Young’s modulus of 2168–2809 MPa for I-WP and 2520 MPa for F-RD.

Surface-based lattice structures have superior mechanical properties [7]; however, it is important to investigate its buckling behavior experimentally and numerically using various geometries columns. The buckling behaviors of cellular structure with circular and elliptical holes and strut-based lattice morphologies have been analyzed in different studies [1,25,26,27,28,29]. However, countable researchers have investigated surface-based lattice structures. Therefore, it is necessary to explore the effect of unit cell morphology and column height on the critical buckling load and post-buckling behavior of surface-based lattice structures. Considering these research gaps, the buckling behavior of surface-based lattice structures have been investigated.

In this paper, the authors aim to investigate and reveal the effect of unit cell shape and height of the column on the critical buckling load constructed from various unit cell of TPMS which is a subset of surface-based lattices. Lattice structures were designed by using nTopology (US) [30] software followed by printing using HP MultiJet fusion [31]. To analyze the critical buckling load, uniaxial compressive tests were performed on samples of various heights and unit cell shape. Furthermore, the experimental results were validated by finite element analysis (FEA) using ANSYS Workbench software [32]. The unit cell size is kept constant for each unit cell for comparison purposes. The effect of the unit cell shape and column height on the buckling properties of surface-based lattice structures was studied, which had not been performed previously. The deformations of the lattice structures, including local and global buckling, were examined after compressive force was applied to the samples.

## 2. Material and Methodology

### 2.1. Design of Unit Cell and Samples

**Figure 1:**
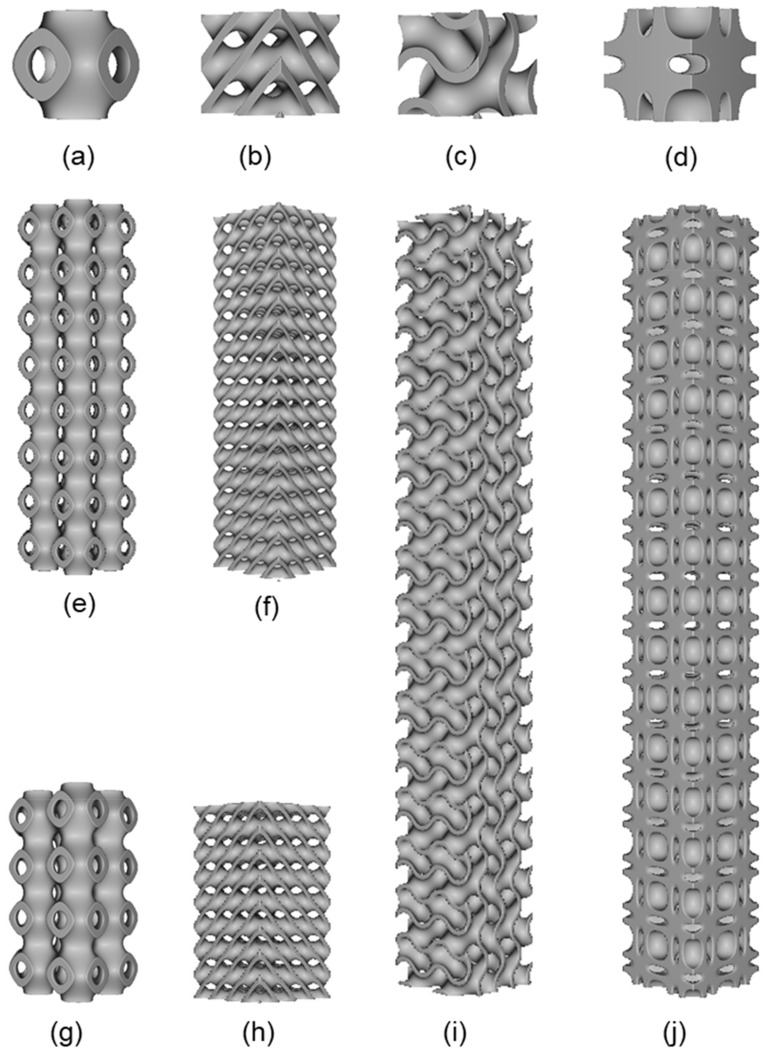
Various design used in this study: (**a**) Schwarz-P unit cell; (**b**) Diamond unit cell; (**c**) Gyroid unit cell; (**d**) Neovius unit cell; (**e**) Schwarz-P (2 × 2 × 8); (**f**) Diamond (2 × 2 × 8); (**g**) Schwarz-P (2 × 2 × 4); (**h**) Diamond (2 × 2 × 4); (**i**) Gyroid (2 × 2 × 16); (**j**) Neovius (2 × 2 × 16).

In this study, four different TPMS based unit cells were selected for buckling study, i.e., Diamond, Gyroid, Neovius and Schwarz-P. Three samples were taken for each unit cell to examine the influence of critical buckling load on column height and unit cell morphology, namely 2 × 2 × 4, 2 × 2 × 8 and 2 × 2 × 16. For this study, unit cell geometry and the entire sample scheme were designed using nTopology [30] software. The dimensions and the design parameters are listed in Table 1. During the design stage, providing the structure with the best surface quality as much as possible was the primary concern. Some of the design samples are shown in Figure 1.

### 2.2. Additive Manufacturing of Samples

**Figure 2:**
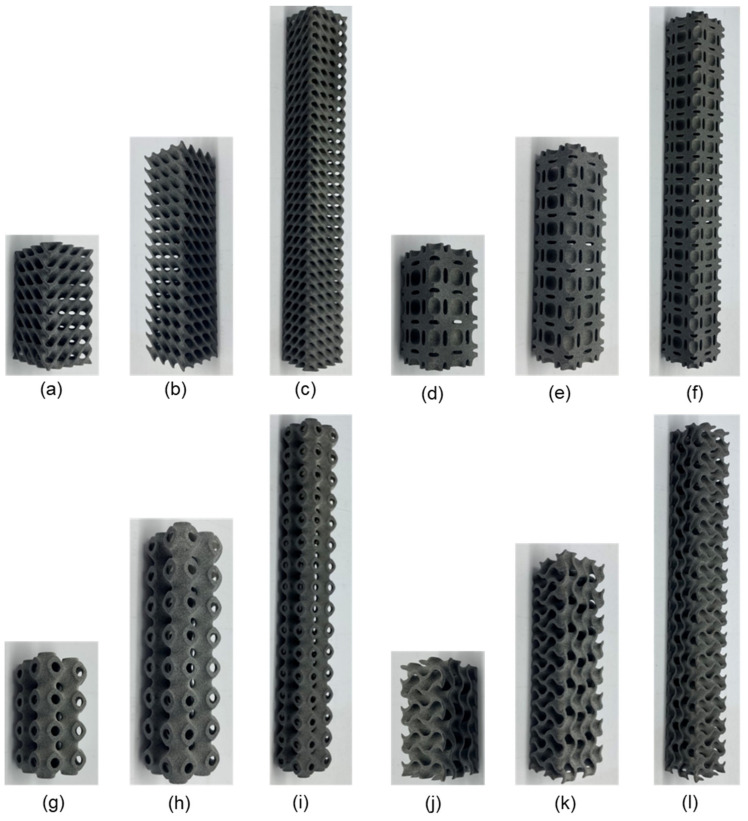
Various sample printed in this study: (**a**) Diamond 2 × 2 × 4; (**b**) Diamond 2 × 2 × 8; (**c**) Diamond 2 × 2 × 16; (**d**) Neovius 2 × 2 × 4; (**e**) Neovius 2 × 2 × 8; (**f**) Neovius 2 × 2 × 16; (**g**) Schwarz-P 2 × 2 × 4; (**h**) Schwarz-P 2 × 2 × 8; (**i**) Schwarz-P 2 × 2 × 16; (**j**) Gyroid 2 × 2 × 4; (**k**) Gyroid 2 × 2 × 8; (**l**) Gyroid 2 × 2 × 16.

Although cellular structures possess several advantageous qualities, their complexity precludes their fabrication without the use of a high-speed additive manufacturing machine. Therefore, a recently developed high-speed AM technology called multi-jet fusion (MJF) was used in this study [31]. The samples were fabricated using Polyamide (PA12) powder, a material that is often utilized in the creation of various injection molded components used in a variety of technical applications. Polyamides offer exceptional qualities, which is why they are employed in a wide variety of sectors, including aircraft, autos, military, medicine, and the environment. Polyamide is a material that is utilized in the manufacture of working parts. [6]. In this research study, the sample of different morphologies was additively manufactured at a vertical position on MJF. Three samples for each lattice morphology were printed to study the buckling behavior, particularly the critical bucking load in uniaxial compression. This printer is capable of printing items with exceptional precision, functionality and surface finish [2]. Additionally, this printer prints at a rate of 4115 cm cube per hour utilizing infrared light as an energy source, and the method achieves a minimum feature size and spacing of 1 mm [6]. Three samples were printed for each lattice morphology. Some printed samples are shown in Figure 2.

### 2.3. Mechanical Testing

The test was conducted using an MTS universal testing machine (MTS Systems Corporation, Eden Prairie, MN, USA) [33]. Compression tests were conducted using a ten-kilonewton load cell at a test speed of two millimeters per minute, according to a well-established and generally recognized protocol for axial compression testing [26,29]. Force and displacement data were continuously recorded using Testworks 4.0 software [34]. Once the slope of the load–displacement curve started decreasing, the test was stopped as this is an indication of buckling. The Young’s modulus of the PA 12 materials, which was previously reported, was also used in this study, while the density and Poisson’s ratio were determined using vendor data [35]. PA 12 material properties are listed in Table 2.

### 2.4. Simulation Framework

The technique of this work depends on experimental investigation as well as simulation using ANSYS Workbench software [32] to identify the buckling behavior of the lattice structures. For the linear buckling investigation, the ANSYS eigenvalue buckling solver is employed to achieve the numerical solution [36]. Numerical analysis of the compressive loaded unit cell was used to determine the efficiency of each design. After efficiency was determined, the optimized design was printed in a 3D printer. The primary objective is to create additively manufactured cellular structures with enhanced functional and mechanical properties. The stress at which a component buckles is determined by its stiffness and not by the strength of its materials. Buckling is a term that refers to a component’s lack of stability and is often unrelated to material strength. This loss of stability often happens within the material’s elastic range. Different differential equations regulate the two phenomena. Buckling failure is primarily defined by a loss of structural stiffness and is modeled using a finite element eigenvalue–eigenvector Equation (1) solution:

|K + λ_m_ K_F_ |δ_m_ = 0, (1)

where λ_m_ is the buckling load factor (BLF) for the m-th mode, K_F_ is the additional “geometric stiffness” due to the loading stresses, F, and δ_m_ is the associated buckling displacement shape for the m-th mode. While the load’s geographical distribution is critical, its proportional amount is not. The computation of buckling produces a multiplier that scales the magnitude of the increase or decrease the load to the point where buckling occurs [37].

**Figure 3:**
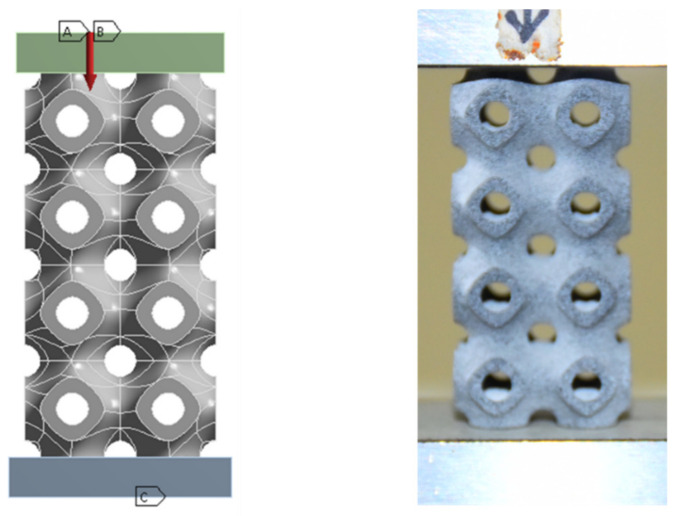
(**Left**): FEA setup showing load application and boundary conditions (A, B and C are load, remote displacement, and fix support respectively). (**Right**): Setup for uniaxial compression.

As in experiment, the following boundary conditions are applied in ANSYS for simulation: At the top end, the compressive load is applied, and at the bottom end, fix support is applied. Fix support at bottom end means that the boundary condition was assumed to be restrained in both lateral and longitudinal displacements as well as rotation. The boundary condition at the top of the cellular column was assumed to have zero lateral displacements (x = z = 0), free longitudinal displacements (y ≠ 0) and no rotation. Boundary conditions are shown in Figure 3.

## 3. Results and Discussion

A total of 12 samples were selected for this research to explore the influence of unit cell shape and column height on critical buckling load. For each sample, three specimens were printed and evaluated using an MTS universal testing system with displacement control. After testing three specimens of each design configuration, average findings are determined and provided in this section.

### 3.1. Buckling of 2 × 2 × 4 Samples

**Figure 4:**
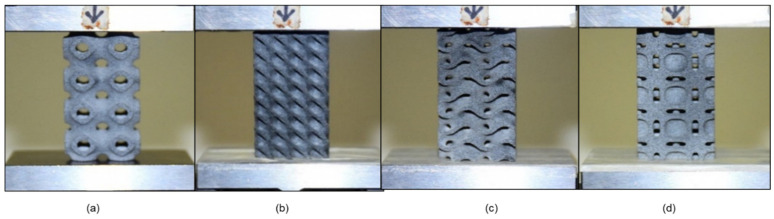
Configuration samples at 2 × 2 × 4 compressed to 0% compression. (**a**) Schwarz-P, (**b**) Diamond, (**c**) Gyroid and (**d**) Neovius.

**Figure 5:**
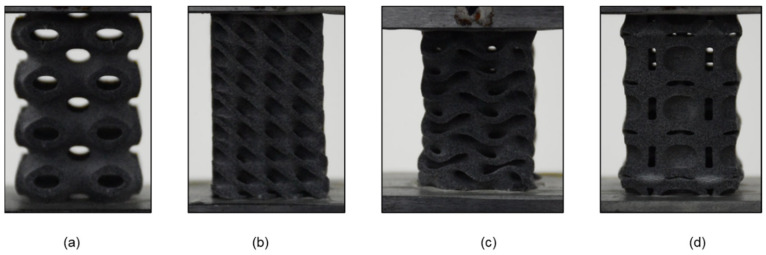
Configuration samples at 2 × 2 × 4 at uniform deformation. (**a**) At this stage, the Schwarz-P sample shows deformation in holes as the shape of the hole’s changes from circular to parabola. (**b**) Diamond design deforms in the share plane. (**c**) Gyroid samples show layer by layer deformation and act like spring. (**d**) Neovius structures show deformation in stress concentration points from where the crack began.

**Figure 6:**
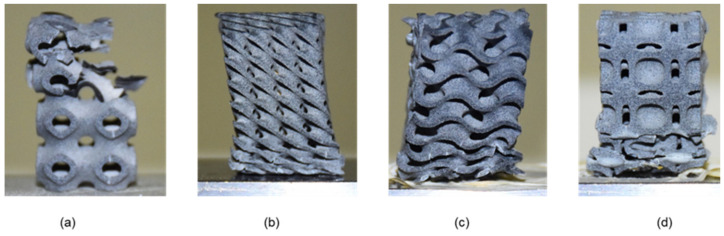
Samples with deformation in the 2 × 2 × 4 arrangement. Design failures occur in the following ways: (**a**) Schwarz-P design fails due to the propagation of crack from stress concentration points; (**b**) Diamond design fails in the same share plane where uniform deformation occurred. (**c**) Gyroid design fails at the bottom end where the load is transferred and (**d**) Neovius design fails due to the propagation of crack from stress concentration points.

**Figure 7:**
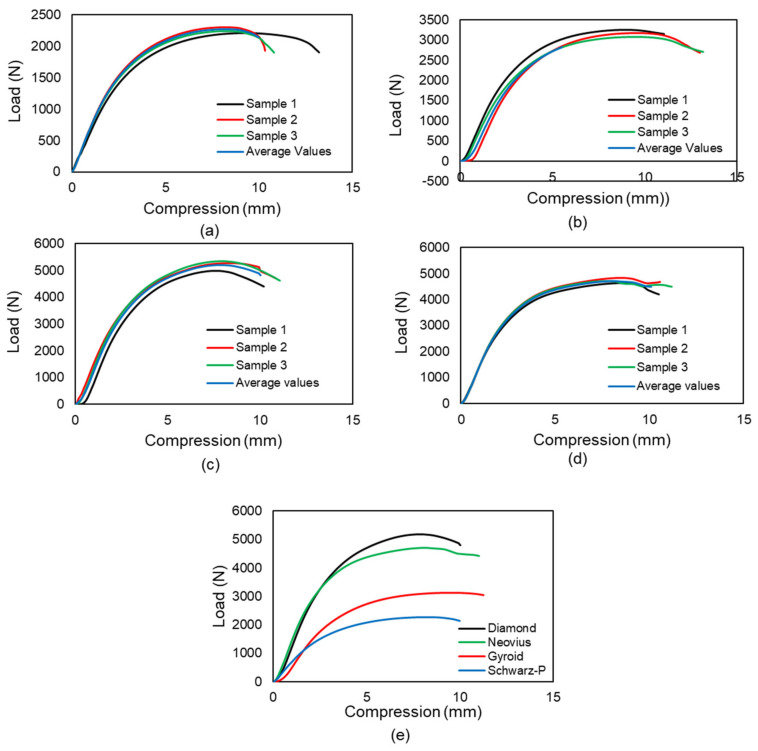
Load-compression graphs. The graphs a, b, c and d indicate that the three specimens of each sample exhibit the same behavior in the compression test, which suggests that printing performance is satisfactory. Average values are taken for future calculations: (**a**) Schwarz-P samples, (**b**) Gyroid samples, (**c**) Diamond samples and (**d**) Neovius samples. (**e**) Four sample average values comparison. The Diamond design exhibits the maximum buckling resistance, whereas Schwarz-P design displays the least resistance to buckling.

The samples were subjected to a uniaxial compression test to determine the critical buckling load. The testing was conducted using an MTS universal testing machine (MTS Systems Corporation, Eden Prairie, MN, USA). Because the Diamond design includes a share plane, the sample demonstrates buckling in that plane direction. The Gyroid design exhibits homogeneous deformation at the start; the sample compresses layer by layer but deforms at the bottom end with bigger displacements. The Gyroid structures behave similarly to springs because the intermediate walls transmit the weight to the bottom end. The cells of the middle unit remain unaltered. The Neovius structure deforms from top to bottom, preserving the integrity of the center unit cells. There is a stress concentration point, and the sample begins to fail at this point, propagating the crack until the sample fractures entirely. The Schwarz-P sample deformed uniformly at low displacement as the circular holes transformed to parabola forms, and then it buckled at the top and bottom ends after substantial displacement. In comparison to the end unit cells, the intermediate unit cell was less distorted. Because to the outer shell carrying the load, the outer shells buckled, and due to their low relative density, they exhibit the least resistance to buckling. Figure 4 illustrates the 2 × 2 × 4 samples compressed to 0%. The uniform deformation of 2 × 2 × 4 samples is shown in Figure 5, while the deformed 2 × 2 × 4 samples are depicted in Figure 6. Figure 7 illustrates load compression graphs.

### 3.2. Buckling of 2 × 2 × 8 and 2 × 2 × 16 Samples

**Figure 8:**
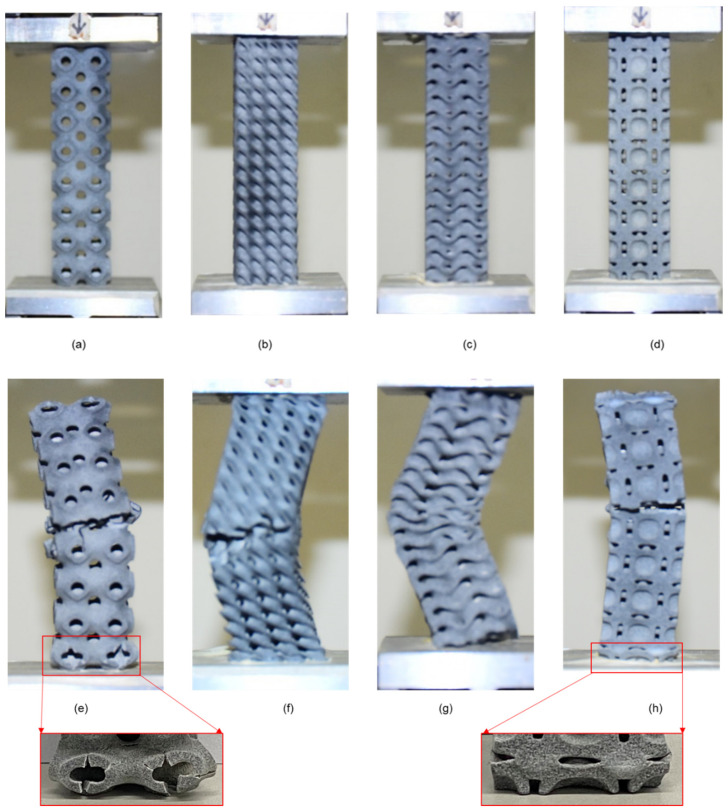
Samples of the 2 × 2 × 8 setup reduced to 0 percent compression: (**a**) Schwarz-P, (**b**) Diamond, (**c**) Gyroid and (**d**) Neovius. Buckled samples of the 2 × 2 × 8 arrangement. (**e**) The Schwarz-P design displays global deformation in the center and local deformation at the bottom end, where the design’s hole is distorted, as shown in the large view. (**f**) Diamond design demonstrates global buckling in the center whereas no local deformation is seen. (**g**) Gyroid design displays no local deformation. (**h**) The Neovius design reveals local deformation at the bottom end, which may be observed in large view.

**Figure 9:**
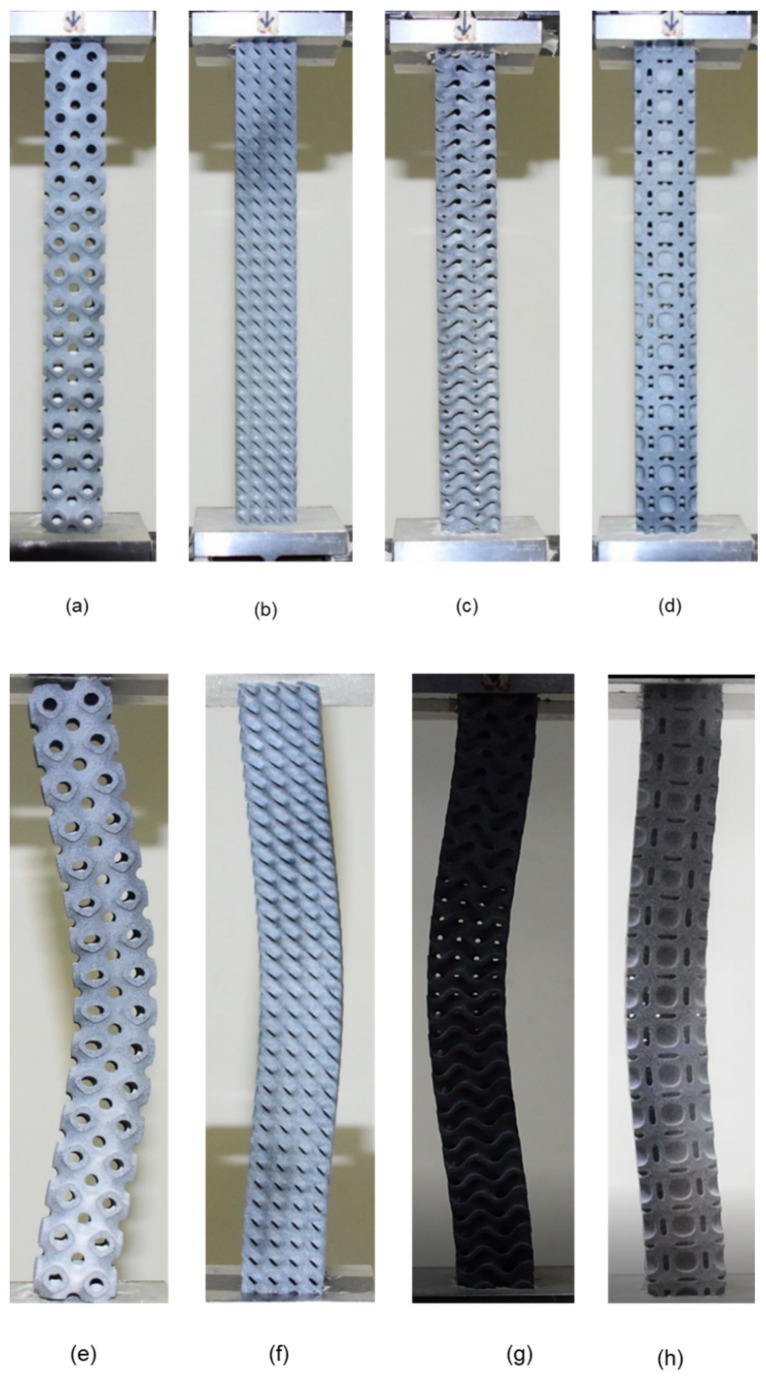
Samples of the 2 × 2 × 16 configuration reduced to 0 percent compression. (**a**) Schwarz-P, (**b**) Diamond, (**c**) Gyroid and (**d**) Neovius. Sample of the 2 × 2 × 16 configuration indicates all samples are buckled only globally; no local buckling is detected. (**e**) Schwarz-P, (**f**) Diamond, (**g**) Gyroid (**h**) Neovius.

**Figure 10:**
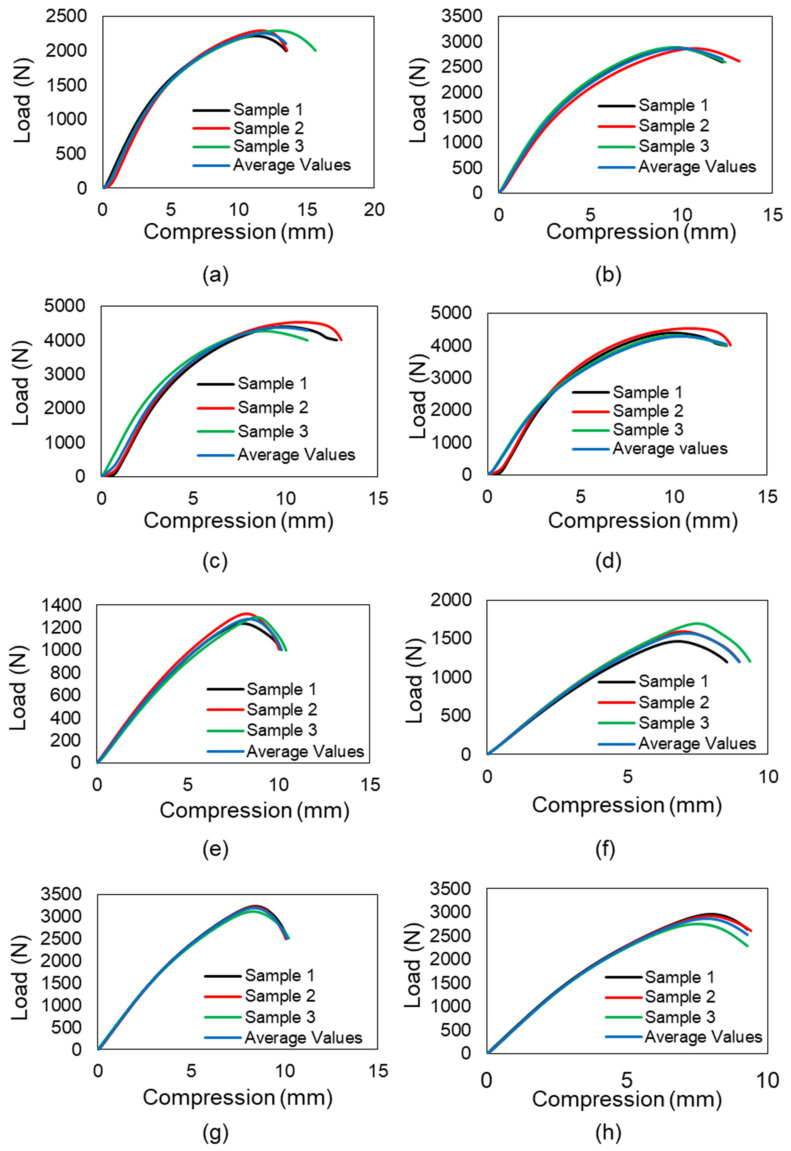

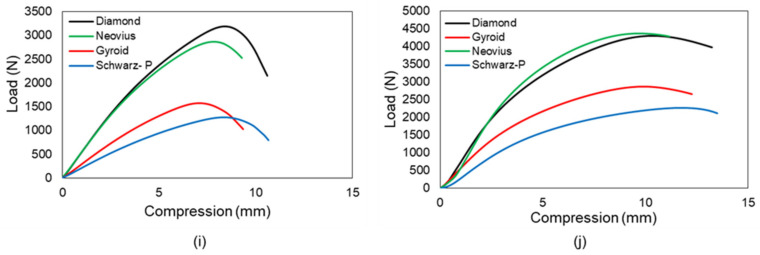
Graphs of load compression. The graphs a, b, c, d, e, f, g and h reveal that the compression test results for the three specimens of each sample are identical, indicating that printing performance is excellent. For subsequent calculations, average values are used. Samples at 2 × 2 × 8: (**a**) Schwarz-P, (**b**) Gyroid, (**c**) Diamond and (**d**) Neovius. Samples at 2 × 2 × 16: (**e**) Schwarz-P, (**f**) Gyroid, (**g**) Diamond and (**h**) Neovius. (**i**) Comparison of four 2 × 2 × 16 samples reveals a significant variance in their critical buckling loads average values. (**j**) Comparing the average results of four 2 × 2 × 8 samples reveals that Diamond and Neovius exhibit roughly the same load, but Diamond’s critical buckling load is the greatest, whilst Schwarz-P structures shows least value.

Both the Gyroid and Diamond samples buckle from the middle, with the Diamond buckle in the direction of the share plane. Thus, because the Neovius and Schwarz-P designs lack an inner wall, the entire load is carried by the outer shell, and these samples exhibit less buckling resistance. In Schwarz-P, the outer shells cause buckling, and the sample fails in the thin neck region. At stress concentration points, Neovius samples buckle. Schwarz-P and Neovius 2 × 2 × 8 samples exhibit local deformations at the top and bottom ends, whereas Diamond and Gyroid 2 × 2 × 8 samples exhibit no local deformation. There is no evidence of local deformation in any of the designs’ 2 × 2 × 16 columns. The buckled samples of 2 × 2 × 8 and 2 × 2 × 16 specimens are shown in Figure 8 and Figure 9, respectively. Figure 10 illustrates load-compression graphs. The local deformation of Schwarz-P and Neovius samples can be seen in Figure 8e,h, where the Schwarz-P type exhibited local deformations at the bottom end as the holes deform, while the Neovius sample exhibited local deformations of the stress concentration points at the bottom end.

### 3.3. Comparison of Critical Buckling Load

#### 3.3.1. Effect of Lattice Morphologies

**Figure 11:**
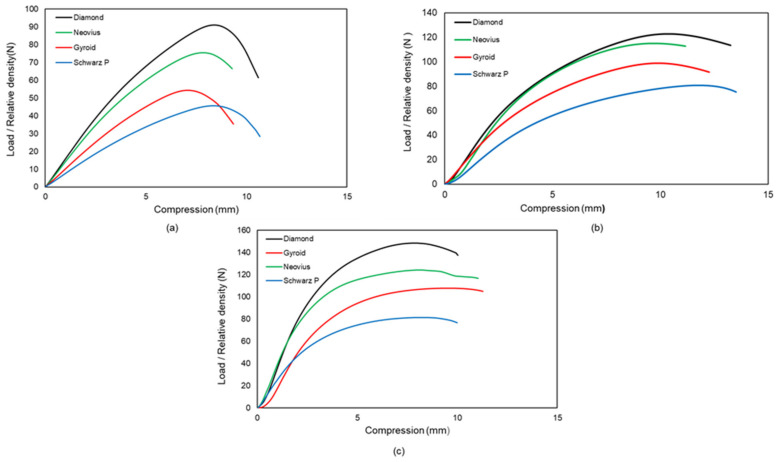
Regardless of relative density, the effect of lattice morphologies on critical buckling load. (**a**) Comparison of 2 × 2 × 16 configurations. (**b**) Comparison of 2 × 2 × 8 configurations. (**c**) Comparison of 2 × 2 × 4 configurations. The same result holds true for all three graphs: Diamond has the highest value, followed by Neovius and Gyroid, respectively, while Schwarz-P has the lowest value.

The investigation of the influence of lattice morphologies on critical buckling loads is an essential objective of this work. It is the relative density of lattice structures that is most critical to the mechanical characteristics of these structures. According to this definition, the relative density is the ratio between the visible lattice structure density and the mass density of the solid substance that makes up the lattice structure. Relative density is a common unit of measure for lattice structure mechanical characteristics [38,39]. For example, the critical buckling load is highly dependent on the relative density of the material. Because the relative density of the samples used in this study varied, the ratio between critical buckling load and relative density was used as a point of comparison in this study. Regardless of relative density, the Diamond samples demonstrate the greatest resistance to buckling, followed by the Neovius and Gyroid samples, and the Schwarz-P samples demonstrate the least resistance. It is the uniform material distribution and large load bearing area of Diamond unit cells that provide its superior buckling resistance when compared to other types of unit cells. Diamond structures have internal walls that can withstand compression loads as well, as opposed to Schwarz-P structures, which can withstand the entire compression load due to the outer shells. Gyroid unit cells exhibit uniform layer-by-layer deformation due to the fact that this unit cell transmits the load from top to bottom through internal walls, as opposed to Neovius unit cells, which exhibit deformations beginning at stress concentration points and propagating until fractures. Figure 11 depicts the load/relative density-compression graphs derived from the data.

#### 3.3.2. Effect of Column Height

**Figure 12:**
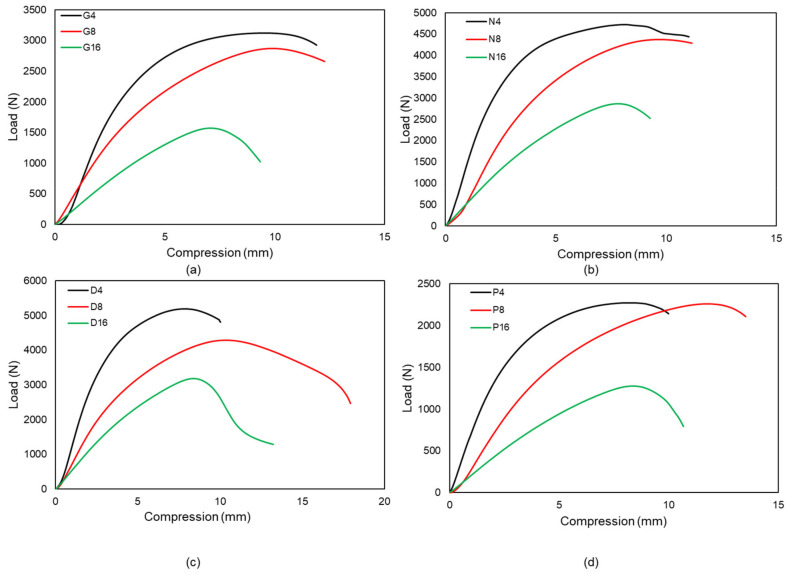
Height’s influence on critical buckling load. The letters D, G, N, and P are used in Graphs to represent Diamond, Gyroid, Neovius and Schwarz-P designs, respectively. The number 4 denotes a 2 × 2 × 4 arrangement, whereas the number 8 denotes a 2 × 2 × 8 configuration and 16 denotes a 2 × 2 × 16 configuration. (**a**) The Gyroid design exhibits a considerable reduction in G8 and G16 of critical buckling load compared to G4 and G8. (**b**) The Neovius design exhibits a considerable reduction in N8 and N16 of critical buckling load when compared to N4 and N8. (**c**) The critical buckling load of Diamond structures decreases uniformly with height. (**d**) Because local deformation is prevalent in Schwarz-P, it may be exploited in short column applications.

In long columns, buckling occurs when the large transverse deformation is exhibited visibly in the structure and resistance to deformation rapidly decreases. This failure is caused by the compression of the column [40]. The critical buckling load of lattice structures depends on various parameters, such as the length of the column, wall thickness, relative density, tessellation, the presence of the column inside the structure and lattice pore size [21,41,42,43,44]. Among the designs employed in this research, Diamond samples exhibit a consistent drop in critical buckling load with increasing height; hence, the Diamond design may be used for columns when height is necessary. Diamond samples display local deformations in 2 × 2 × 4, but only global buckling is detected in 2 × 2 × 16 designed due to the fact that the sample critical buckling load reduces equally with height. Schwarz-P unit cell design for 2 × 2 × 4 and 2 × 2 × 8 samples shows nearly the same critical load because local deformation is prominent in both samples; hence, this kind of structure is best suited in short columns as the Schwarz-P’ unit cell’s relative density is likewise low compared to Neovius. For Gyroid and Neovius designs, the difference between 2 × 2 × 4 and 2 × 2 × 8 is quite modest as opposed to the difference between 2 × 2 × 8 and 2 × 2 × 16, as seen in Figure 12a,b, respectively. For the comparison of height to critical buckling load, the load versus compression graph is provided in Figure 12 for all samples evaluated in this research study.

### 3.4. Validation Using Simulation Results

The material properties used in FEA are listed in Table 2, while the simulation framework section describes the methods for performing the eigenvalue buckling simulation and the boundary conditions used. This analysis makes use of a 1 mm mesh size for the Tet10 elements in the simulation of all lattice structures. Table 3 compares the experimental and simulation results for eight design lattice structures with identical unit cell sizes where FEA and experimental data agree well apart from these. Due to the high computational requirements, the other four designs are not simulated. Geometric imperfections, variability in load and boundary conditions, material properties and surface thickness variability all contribute to the discrepancy between experimental and FEA results [43,45,46].

The Euler buckling formula can be used to theoretically predict the critical buckling load. The end restrictions on Euler long column buckling are quite important. Several examples of end restraints and the corresponding k value are possible, which may be utilized for both the limiting slenderness ratio and the buckling load. Euler’s critical buckling force (F_Euler_) is defined in Equation (2).

F_Euler_ = k π^2^ E I/L^2^ (2)

The radius of gyration, r, is defined in Equation (3).

r = (I/A)^1/2^ (3)

Euler’s critical buckling force formula is defined in Equation (4) after substituting Equation (3) in Equation (2).

F_Euler_ = k π^2^ E A/(L/r)^2^ (4)

where I and A are the area moment of inertia and area of the cross-section, respectively, while E is the elastic modulus. L/r is slenderness ratio” where L denotes the length of the component. k is a constant that depends on the restraints of the two ends of the column [6,37].

**Figure 13:**
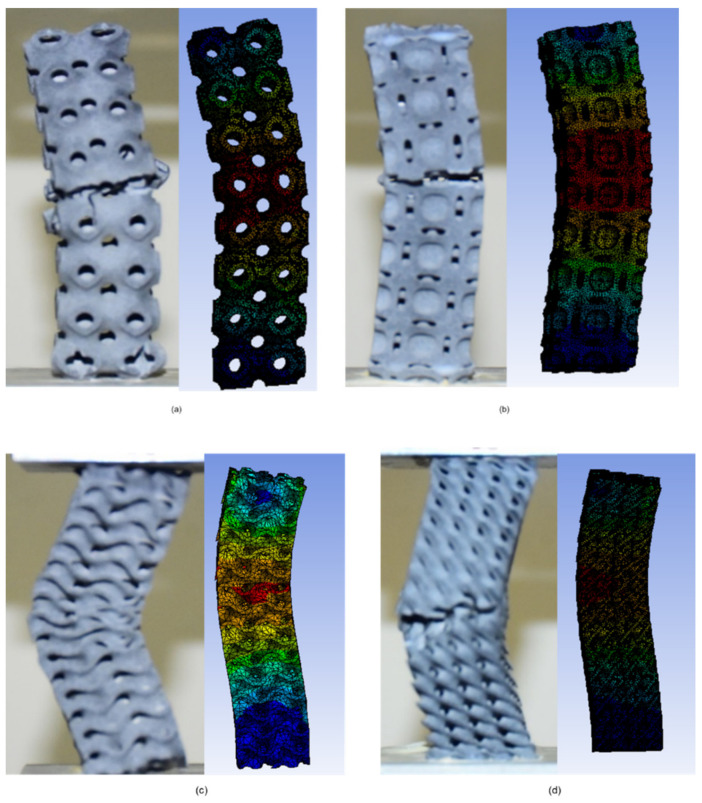
Numerical and experimental patterns of buckling behavior for uniaxial compressive samples of various morphologies. The figures reveal that the experimental and FEA results show good agreement. Samples with 2 × 2 × 8 configuration: (**a**) Schwarz-P, (**b**) Neovius, (**c**) Gyroid and (**d**) Diamond.

**Figure 14:**
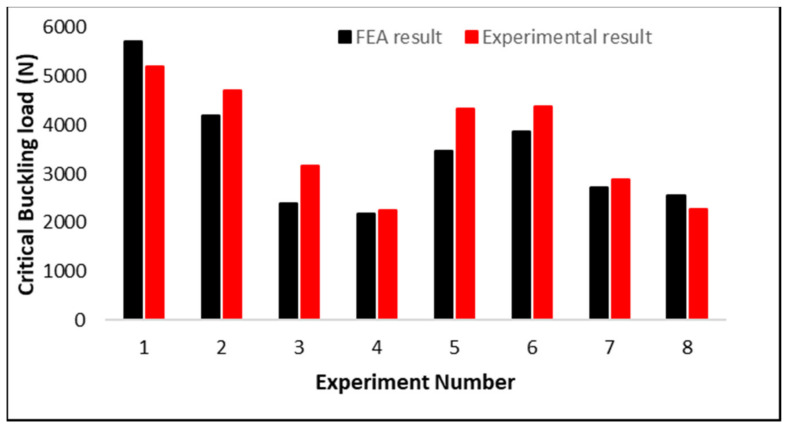
Comparison of experimental and simulation results as indicated in Table 3.

Applying the load at the precise center of the design is highly critical; however, under experimental boundary circumstances, the load may not be totally eccentric. Figure 13 demonstrates the comparable pattern of experimental and FEA behavior of the designs, which indicates that the linear eigenvalue buckling analysis may approach the same behavior of the buckling, as shown in experimental data. Based on these eight numerical simulations, the additional four experiments may be estimated to demonstrate excellent agreement with FEA. The comparison is also described in Figure 14. The critical buckling (bifurcation) load of structures was estimated using eigenvalue buckling analysis. A linear perturbation approach is used in the analysis. It may be used to simulate measured initial overall and local geometric defects, as well as to investigate a structure’s imperfection sensitivity when measurements are unavailable. To determine the critical buckling loads of stiff structures, eigenvalue buckling is commonly utilized (classical eigenvalue buckling). Rather than bending, stiff structures transmit their design loads predominantly by axial or membrane action. Prior to buckling, they normally exhibit relatively little deformation. The specimens in this study were printed using MJF, and their properties are not fully isotropic due to various reasons such as porosity, build orientation, the effect of post-processing, residual powder and different printer constraints due to which the parts possess some non-linear behavior. Most of these cannot be considered during finite element analysis. Due to this, there are some variations between experimental and simulation results [47,48].

## 4. Conclusions

In this study, twelve columns of four different triply periodic minimal surface unit cells of the same size were successfully printed using multi-jet fusion technology. The investigation of the critical buckling load is performed by performing a uniaxial compression test. Among these four different unit cells, the Diamond design showed the maximum resistance to buckling, followed by Neovius and Gyroid, respectively, while Schwarz-P showed the least resistance to buckling. The effect of height column on critical buckling load is also investigated, which reveals that Diamond design shows a uniform decrease with height, while in the Schwarz-P sample, the samples 2 × 2 × 4 and 2 × 2 × 8 show plastic deformation dominancy. Diamond and Gyroid samples show buckling in the share plane and layer-by-layer compression, respectively, while in Schwarz-P and Neovius designs, crack propagation from the stress concentration points causes failure.

It is concluded that the Diamond design showed the highest critical buckling load irrespective of relative density due to uniform stress distribution and more load bearing cross-section area. The Schwarz-p structure show the least critical buckling load due to stress concentration points. The Neovius sample has high load bearing areas due to the unit cell joined in the inner part, but at the outer shells, there are stress concentration points. Overall, the unit cell morphologies greatly affect the critical buckling load irrespective of relative density, which enables the researchers to select the specific lattice structure for specific applications according to the required load applications.

An FEA analysis was performed to validate experimental results, which showed a resemblance with experimental results. Although the buckling pattern is the same for both experimental and FEA, there are considerable variations in the specimen of specific lattice structure design due to changes in material properties during printing, build orientation and residual powder; moreover, the structure was not truly isotropic, but it may be approximated as isotropic. Further analysis needs to be performed by designing different unit cells size columns of the same unit cells and studying the effect of unit cell size in terms of critical buckling loads.

## Figures and Tables

**Table 1:** The dimension and the design parameter of sample used in this study.

Geometry	Sample	Unit Cell Size (mm)	Thickness (mm)	Sample Height (mm)	Sample Width (mm)	Sample Breadth (mm)	Relative Density of Designed Parts (%)	Relative Density of Fabricated Parts (%)
	2 × 2 × 4			48				36
Diamond	2 × 2 × 8			96			35	33
	2 × 2 × 16		1.8	192				39
	2 × 2 × 4			48				30
Gyroid	2 × 2 × 8	12		96	24	24	29	31
	2 × 2 × 16			192				30
	2 × 2 × 4			48				39
Neovius	2 × 2 × 8			96			38	41
	2 × 2 × 16		1	192				40
	2 × 2 × 4			48				27
Schwarz-P	2 × 2 × 8			96			28	29
	2 × 2 × 16			192				27

**Table 2:** PA 12 material properties.

Density (g/cm^3^)	Young’s Modulus (MPa)	Poisson’s Ratio
1.01	1250	0.33

**Table 3:** Comparison of FEA and experimental results.

Experiment No.	Geometry	Sample	FEA Result for Critical Buckling Load (N)	Experimental Result for Critical Buckling Load (N)	Error (%)
1	Diamond		5708	5193	9.93
2	Neovius	2 × 2 × 4	4196	4708	10.86
3	Gyroid		2390	3163	24.40
4	Schwarz P		2174	2250	3.35
5	Diamond		3470	4340	20.04
6	Neovius	2 × 2 × 8	3877	4387	11.61
7	Gyroid		2715	2880	5.70
8	Schwarz P		2555	2271	12.52

## Data Availability

Not applicable.
